# Unveiling coagulation dysfunction in patients with COVID-19: a retrospective analysis

**DOI:** 10.25122/jml-2024-0166

**Published:** 2024-09

**Authors:** Ahmed Ali Jerah, Abdullah Farasani, Hisham Abu-Tawil, Hadi Kuriri, Manal Mohamed Elhassan Taha, Siddig Ibrahim Abdelwahab, Osama Albasheer

**Affiliations:** 1 Department of Medical Laboratory Technology, Faculty of Applied Medical Sciences, Jazan University, Jazan, Saudi Arabia; 2 Biomedical Research Unit, Medical Research Center, Jazan University, Jazan, Saudi Arabia; 3 Department of Clinical Laboratory and Blood Bank, King Faisal Medical City for Southern Regions, Abha, Saudi Arabia; 4 Department of Clinical Laboratory and Blood Bank, Samtah General Hospital, Samtah, Saudi Arabia; 5 Medical Research Centre, Jazan University, Jazan, Saudi Arabia; 6 Family and Community Medicine Department, College of Medicine, Jazan University, Jazan, Saudi Arabia

**Keywords:** COVID-19, coagulation, thromboplastin, prothrombin, mortality

## Abstract

Coagulation dysfunction has emerged as a significant aspect of COVID-19 pathophysiology, with abnormal coagulation parameters observed in severe cases. This study aimed to investigate the predictive value of coagulation parameters, including prothrombin time (PT), activated partial thromboplastin time (PTT), and international normalized ratio (INR) for mortality in patients with COVID-19. A retrospective analysis was conducted on a cohort of patients diagnosed with COVID-19. Coagulation parameters, including PT, PTT, and INR, were measured upon admission. Receiver operating characteristic (ROC) curve analysis was performed to evaluate the predictive performance of these parameters. Sensitivity and specificity were calculated, and the area under the curve (AUC) values were determined. The analysis included 156 patients diagnosed with COVID-19. The *t*-test revealed a significant difference (*P* < 0.05) in PTT, PT, and INR. PTT demonstrated the highest predictive performance, with an AUC value of 0.68, indicating superior discrimination compared with PT and INR. PTT exhibited a sensitivity of 83% and a specificity of 46% for identifying deceased patients. These findings suggest that PTT may serve as a valuable prognostic marker of mortality risk in patients with COVID-19. Coagulation indicators, particularly PTT, predicted COVID-19 mortality. Monitoring coagulation markers may help stratify the risk and guide treatment. Further research and validation studies are needed to corroborate these findings and to establish the clinical importance of coagulation markers in COVID-19 therapy. COVID-19 coagulation dysfunction mechanisms must be understood in order to design targeted therapies to reduce thrombotic consequences.

## INTRODUCTION

The advent of the SARS-CoV-2 virus in China in 2019 has led to a global health crisis known as the COVID-19 outbreak. The virus has rapidly disseminated globally, leading to millions of confirmed cases and a substantial number of fatalities [1,2]. COVID-19 exhibits a spectrum of clinical presentations ranging from minor respiratory symptoms to severe respiratory distress and even fatality. Identifying factors that can help predict disease severity is crucial for effective patient management and resource allocation [3,4].

Coagulation dysfunction has emerged as an important aspect of COVID-19 pathophysiology. Several studies have reported evidence of abnormal coagulation parameters and a higher incidence of thrombotic complications in severe COVID-19 cases [1,5]. Dysregulated coagulation pathways are believed to contribute to the development of microvascular thrombosis, disseminated intravascular coagulation (DIC), and venous thromboembolism in COVID-19 patients [1,2,5]. Identifying coagulation dysfunction as a prominent feature of severe COVID-19 has important clinical implications. Assessing coagulation markers can help categorize risk and promptly detect individuals more likely to have disease progression and consequences [5,6]. This information can guide therapeutic decisions, including initiating anticoagulant therapy, which has shown potential benefits in reducing thrombotic events and improving patient outcomes [7,8].

In line with the importance of understanding coagulation dysfunction in COVID-19, several retrospective analyses have been conducted to investigate coagulation parameters in infected individuals [9]. These analyses aimed to identify potential biomarkers that could aid in predicting disease severity and improving patient outcomes [10,11]. One retrospective analysis by Tang *et al*. examined coagulation parameters in 183 COVID-19 patients [12]. The study found that elevated D-dimer levels, fibrin degradation products, and prolonged prothrombin time were associated with higher mortality risk. Another retrospective study by Zhang *et al*. analyzed coagulation profiles in patients with COVID-19 [13]. They found that increased D-dimer levels were strongly linked to the severity of the disease, and individuals with higher D-dimer levels were more likely to die.

The objective of our study was to investigate coagulation parameters, specifically partial thromboplastin time (PTT), international normalized ratio (INR), and prothrombin time (PT), in patients diagnosed with COVID-19. We aimed to evaluate the potential associations between these coagulation parameters and disease severity to provide insights into the role of these parameters as predictors of clinical outcomes in COVID-19.

## Material and Methods

### Demographics and study location

This study retrospectively analyzed 156 patients (83 men and 73 women). The median patient age was 54.73 years. The study was conducted at a general hospital in Samtah, Jazan, from April 2020 to October 2021. The essential demographic information and biochemical markers of the patients were obtained from electronic medical records. Due to the retrospective nature of this study, there was no direct interaction with patients, guaranteeing the rigorous preservation of their privacy and confidentiality. COVID-19 diagnosis was validated by reverse transcriptase-polymerase chain reaction (RT-PCR) analysis [3]. This analysis was conducted on nasopharyngeal swab specimens following the established standards of the Center for Disease Control and Prevention in Saudi Arabia [14].

### Criteria for inclusion and exclusion

Initially, a group of 2010 individuals who tested positive for COVID-19 and underwent screening were considered for inclusion in the study. The inclusion criteria in the study were cases of COVID-19 confirmed through laboratory testing and with complete hematological profiles available at the time of admission. The analysis excluded patients with prior hematological abnormalities or insufficient medical records. Electronic health records offer vast data, including demographic information, clinical symptoms, laboratory findings, illness severity, and treatment outcomes. After applying these criteria for inclusion, 156 patients who fulfilled all relevant requirements were included in the study.

The study adhered rigorously to the ethical criteria specified in the Declaration of Helsinki and other relevant regulations [15]. All data were anonymized and securely stored to protect patient privacy and confidentiality. Since this was a retrospective analysis, patient consent was not required, as there was no direct interaction with patients or risk of harm. The research team maintained the integrity of the study and ensured that findings were presented while preserving participants' anonymity. Ethical approval was obtained to ensure full compliance with ethical standards, safeguarding patients' rights and well-being throughout the research.

### Quantitative indicators

The retrospective analysis included sex, age, weight, height, body mass index (BMI), mortality, and coagulation profile. The main aim of this study was to assess the coagulation parameters of patients with COVID-19 and determine any significant correlations between these parameters and illness outcomes.

### Data analysis

The data were analyzed using IBM-SPSS version 23.0 (USA), employing descriptive and inferential statistical methods. Continuous variables were presented as means or ranges, depending on their distribution, while categorical variables were summarized as counts and percentages. Categorical variables were summarized by calculating the number of occurrences and expressing them as percentages. The normality of the distribution of continuous variables was evaluated using the Kolmogorov-Smirnov test. Independent sample *t*-tests were used to assess disparities between the mild and severe groups. The receiver operating characteristic (ROC) curve and the corresponding area under the curve (AUC) were computed to compare each parameter [16]. The criterion for statistical significance was set at a *P* value of less than 0.05.

## RESULTS

The demographic and clinical characteristics of the study population are summarized in Table 1. The gender distribution showed that 83 individuals (53.2%) were men and 73 (46.8%) were women. In terms of age, 42 individuals (26.9%) were less than 40 years old, 30 (19.2%) were between 40 and 60 years old, and the majority (84 individuals, 53.8%) were over 60 years old. The categories of BMI indicated that 14 individuals (9.2%) were classified as underweight (< 18.5), 56 individuals (36.6%) had a normal BMI (18.5 - 24.9), 58 individuals (37.9%) were overweight (25 - 29.9), and 25 individuals (16.3%) were classified as obese (> 30). Regarding comorbidity status, 62 individuals (39.7%) had no comorbidities, whereas 94 individuals (60.3%) had comorbidities. The mortality data revealed that 133 individuals (85.3%) were still alive, whereas 23 individuals (14.7%) had passed away. This table provides a comprehensive overview of the demographic and clinical characteristic distributions within the study population.

**Table 1:** Demographic and clinical characteristics

Variables	*n* (%)
**Gender**	
Male	83 (53.2)
Female	73 (46.8)
**Age**	
Less than 40 yrs	42(26.9)
40 - 60 yrs	30(19.2)
More than 60 yrs	84(53.8)
**Body mass index**	
Underweight (< 18.5)	14(9.2)
Normal (18.5 - 24.9)	56(36.6)
Overweight (25 - 29.9)	58(37.9)
Obese (> 30)	25(16.3)
**Comorbidity**	
No comorbidity	62(39.7)
Comorbidity	94(60.3)
**Mortality**	
Survivor	133 (85.3)
Deceased	23(14.7)

Table 2 compares the coagulation parameters of deceased and surviving participants using the Student's *t*-test. This study examined three coagulation parameters: prothrombin time (PT), INR, and PTT. The table displays the mean value with standard error of the mean (S.E.M.), the accompanying *t*-value, and the significance for each parameter. In terms of PT, the mean PT value for survivors was 15.56 ± 0.59 and 20.21 ± 2.52 for deceased participants. A *t*-value of 2.70 with a significance threshold of 0.008 indicates a significant difference in PT between the two groups. In terms of INR, the mean INR value for survivors was 1.24 ± 0.04 and 1.66 ± 0.25 for deceased participants. A significant difference in INR between the two groups was indicated by a *t*-value of 2.77 with a significance level of 0.007. The mean PTT value for surviving participants was 33.52 ± 1.15 and 42.86 ± 5.29 for deceased participants. A *t*-value of 2.69 with a threshold significance of 0.008 indicates a significant difference in PTT between the two groups. Table 2 shows significant changes in coagulation parameters between deceased and surviving subjects, indicating potential links between these parameters and clinical outcomes.

**Table 2:** Comparison of coagulation parameters between survivors and non-survivors using Student's *t*-test

Parameters	Clinical outcome	Mean ± S.E.M.	*t*-value (significance)
PT (seconds)	Survivor	15.56 ± 0.59	2.70(0.008)
	Deceased	20.21 ± 2.52	
INR (seconds)	Survivor	1.24 ± 0.04	2.77(0.007)
	Deceased	1.66 ± 0.25	
PTT (seconds)	Survivor	33.52 ± 1.15	2.69(0.008)
	Deceased	42.86 ± 5.29	

PT, Prothrombin time; INR, international normalized ratio; PTT, activated partial thromboplastin time

**Figure 1:**
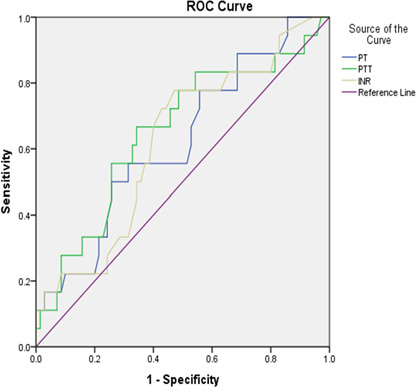
ROC curve analysis of PT, PTT, and INR for predicting mortality in COVID-19 patients

Subsequently, we conducted a more in-depth analysis of PT, PTT, and INR and computed the receiver operating characteristic curve (ROC) and area under the curve (AUC) for these three parameters. Figure 1 and Table 3 demonstrate that PTT had the greatest AUC value of 0.68, whereas the AUC values for PT and INR were 0.64. According to the data in Table 2, PTT demonstrated superior predictive ability for deceased patients, with a sensitivity of 78% and a specificity of 53%.

**Table 3:** The predictive values of PT, PTT, and INR in patients with COVID-19

Parameter	AUC	*P* value	Cut off value	Sensitivity (%)	Specificity (%)
PT	0.64	0.12	14.15	78	45
PTT	0.68	0.12	30.45	83	46
INR	0.64	0.04*	1.160	78	53

ROC, receiver operating characteristic curve; AUC, area under the curve; PT, Prothrombin time; INR, international normalized ratio; PTT, activated partial thromboplastin time. *significant at 0.05

Table 4 presents a multivariate logistic regression analysis examining the relationship between various independent variables and the dependent variable of mortality (0: deceased; 1: survived). The variables included in the study were gender, comorbidity, age, PT sec, INR sec, PTT sec, and BMI. Among these variables, only PT sec was statistically significant in predicting death (*P* = 0.04). Although the female gender had a higher odds ratio for death, the association was not statistically significant (*P* value = 0.13). Comorbidity, age, INR, PTT, and BMI did not show statistically significant associations with mortality, with *P* values greater than 0.05. These results suggest that PT in seconds may significantly predict mortality, while the other variables did not demonstrate a strong relationship with the outcome.

**Table 4:** Multivariate logistic regression analysis of predictors for mortality (0: Deceased; 1: Alive)

Variable	P-value	OR	95% C.I. for OR	
			Lower	Upper
Gender				
Male (Reference group)				
Female	0.13	2.34	0.77	7.06
Comorbidity				
No comorbidity (Reference group)				
Comorbidity	0.44	0.65	0.21	1.97
Age				
Less than 40 yrs (Reference group)				
40 - 60 yrs	0.60	1.66	0.26	10.58
More than 60 yrs	0.97	1.03	0.21	4.98
PT sec	**0.04**	0.77	0.60	0.99
INR sec	0.50	1.47	0.49	4.38
PTT sec	0.40	1.02	0.97	1.07
BMI	0.93	0.99	0.98	1.02

## Discussion

Our study examined the prognostic value of PT, PTT and INR on the severity of COVID-19 infection. We examined the relationship between these coagulation markers and illness severity to determine whether these could predict COVID-19 clinical outcomes. Following the emergence of COVID-19 in Wuhan, this illness has rapidly disseminated globally, prompting the World Health Organization to designate it as a pandemic and public health emergency of international concern [17]. SARS-CoV-2 has been identified as the causative agent of COVID-19. The virus utilizes angiotensin-converting enzyme 2 (ACE2) as one of its receptors for entry into vulnerable cells. SARS-CoV-2, which belongs to the β-coronavirus genus, shares 85% similarity with the bat SARS-like coronavirus [17,18].

Upon infection, disease progression and patient outcomes vary. While the majority of individuals infected with SARS-CoV-2 experience moderate or no symptoms, a subset will progress to severe cases characterized by respiratory failure and potential fatality if not appropriately treated [19,20]. Hence, it is crucial to identify biomarkers such as coagulation markers that can be used to forecast disease progression. These coagulation-related molecules are produced due to the breakdown of cross-linked fibrin during fibrinolysis [21]. Assessing these coagulation-related molecules is crucial for the contemporary evaluation and identification of disseminated intravascular coagulation [22,23]. Chen *et al*. investigated the blood parameters of the first 99 patients hospitalized in Wuhan and found abnormal coagulation parameters such as elevated levels of APTT (6% of the cases), PT (5% of the cases), D-dimer (36% of the cases), interleukin-6, and increased erythrocyte sedimentation rate, as well as C-reactive protein [24]. De la Morena-Barrio *et al*., in a total of 127 hospitalized patients with confirmed COVID-19, observed that the thrombin generation lag-time showed a positive correlation with markers of cell lysis (LDH), inflammation (CRP, IL-6) and coagulation (D-dimer). Conversely, the endogenous thrombin potential (ETP) showed an inverse correlation with D-dimer and LDH and positively correlated with fibrinogen levels [25].

In this retrospective study, we aimed to identify possible biomarkers to differentiate deceased patients from those who survived so that clinicians may respond quickly and provide a more suitable therapeutic scheme for severe patients. We analyzed coagulation function and found that PT, PTT, and INR were higher in the severe group. Table 2 compares the coagulation parameters between deceased and surviving participants. The findings indicate significant differences between deceased and surviving participants for PT (*t* = 2.70, *P* = 0.008), INR (*t* = 2.77, *P* = 0.007), and PTT (*t* = 2.69, *P* = 0.008). These findings suggest that coagulation parameters may be associated with clinical outcomes in patients with COVID-19. In their investigation, Tekle *et al*. noted that severe COVID-19 patients had prolonged baseline PT. They proposed using prolonged baseline PT to predict a worse prognosis and risk stratification [26]. The results of Long *et al*. and Jin *et al*. in China were comparable to this finding [27,28]. On average, PT is 1.9 s longer in fatal COVID-19 cases compared to non-fatal cases and marked progressive prolongation of PT was observed in fatal cases [4,29].

Tang *et al*. found that higher PT and PTT values, as well as significantly increased D-dimer levels, were associated with coronavirus-induced fatalities in their investigation of 183 patients with confirmed coronavirus pneumonia at Tongji Hospital [12]. Similarly, our study found that very elevated PT and PTT levels were highly likely to necessitate intensive care hospitalization.

The results of our in-depth analysis of coagulation parameters, including PT, PTT, and INR, demonstrated that PTT had the highest AUC value of 0.68, indicating better predictive performance than PT and INR, which both had AUC values of 0.64. This finding was similar to the findings of Tekle *et al*., where prolonged PT was observed in 70% of moderate and severe COVID-19 patients, while a prolonged APTT was observed in only 13.7% of severe COVID-19 patients [26]. PTT also exhibited superior sensitivity (78%) and specificity (53%) in identifying deceased patients. These findings suggest that PTT may serve as a valuable prognostic marker of mortality risk in patients with COVID-19. Coagulation abnormalities in severe cases of COVID-19 are well documented, and understanding their implications can guide clinical decision-making and risk stratification. However, further research and validation studies are needed to confirm these results and explore the underlying mechanisms and potential therapeutic implications. Similarly, Maghrabi *et al*. [30], in a multicenter Saudi study of 118 COVID-19 patients, found that coagulation markers may help predict patient outcomes and support timely anticoagulant use to reduce COVID-19-related mortality. These findings are consistent with previous research [31-33] and underscore the importance of monitoring coagulation parameters.

According to Wu *et al*.'s analysis of the Chinese Center for Disease Control and Prevention (CCDC) bulletin, of the 72,314 COVID-19 cases reported, 44,672 (62%) were confirmed, 16,186 (22%) were suspected, 10,567 (15%) were clinically diagnosed, and 889 (1%) were asymptomatic [34]. The severity of the cases was categorized as follows: 81% were mild, 14.5% were severe, and 5% were critical. The study revealed key insights: 2.3% of confirmed patients died, with the mortality rate among non-survivors over 80 years reaching 14.8%, and 49.0% of the critical cases resulted in death. Furthermore, the fatality rate of 50% in critically ill individuals can be regarded as a significant measure of mortality. In contrast, all 23 patients in our study who were admitted to the hospital and received intensive care unfortunately passed away. The increased coagulation parameters observed, such as elevated INR and PT, were instrumental in identifying patients who required intensive or critical care and monitoring their progress in the intensive care unit (ICU). Additionally, there was a notable distinction between ICU survivors and non-survivors in terms of elevated INR and PT levels. The findings of our study can assist clinicians in determining the clinical severity of COVID-19 rather than only the diagnosis. Evaluating INR and PT levels may also assist in determining which patients are likely to need intensive care.

The multivariate logistic regression analysis results suggest that gender, comorbidity, age (40-60 years and more than 60 years), INR sec, PTT sec, and BMI are not independent predictors of death in this study (*P* > 0.05). However, PT sec, representing prothrombin time in seconds, shows a statistically significant association with death (*P* = 0.04). A higher PT sec was associated with a decreased likelihood of death, indicating that longer prothrombin times may indicate better survival outcomes. This study is consistent with previous studies that reported that prolonged prothrombin time leads to poor clinical outcomes in COVID-19 patients [26,27,35]. The lack of significance in the other variables suggests that additional factors not included in the analysis may play a more substantial role in predicting mortality. These findings emphasize the need for further research to validate and generalize the results, as they are specific to the dataset and population studied.

### Limitations

One limitation of this study was the lack of a large sample size, which may affect the generalizability of the findings. The analysis was conducted on a specific group of participants, and the results may not represent a broader population. Additionally, the study focused solely on PT, PTT, and INR as coagulation parameters, neglecting other relevant factors that could influence mortality risk in COVID-19 patients. Clotting times (PT and PTT) are insensitive to the concurrent decrease of inhibitors and only account for drastic reductions in pro-coagulant factor levels, making it difficult to accurately assess the coagulation profile in individuals with coagulopathy. The association of lag-time and ETP with D-dimer might be of great value. Moreover, the study design was retrospective, which introduced potential biases and limitations in the data collection and analysis. Further prospective studies with larger and more diverse populations are required to validate these findings and to provide a more comprehensive understanding of the relationship between coagulation parameters and mortality in COVID-19.

## Conclusion

This study suggests that coagulation parameters, specifically PT, PTT, and INR, may have predictive value for mortality in patients with COVID-19, with PTT demonstrating the highest potential. These findings indicate a potential association between coagulation abnormalities and clinical outcomes in COVID-19, highlighting the dysregulation of coagulation in severe cases. However, this study has limitations regarding sample size and retrospective design. Further research is needed to validate these findings, understand the underlying mechanisms, and integrate coagulation parameters into comprehensive risk assessment models to guide personalized patient care in COVID-19.

## Data Availability

The datasets used and/or analyzed during the current study are available from the corresponding author upon reasonable request.
